# Effect of Preoperative Corticosteroid Injections on Postoperative Risk of Periprosthetic Joint Infection and Revision Surgery After Total Shoulder Arthroplasty: A Systematic Review and Meta-Analysis

**DOI:** 10.7759/cureus.75473

**Published:** 2024-12-10

**Authors:** Muzammil Akhtar, Daniel I Razick, Anand Dhaliwal, Kaitlyn Guadagno, Osamah Baig, Jimmy Wen, Mustafa Jundi, Dawnica Nadora, Denise Nadora, Eric Huish

**Affiliations:** 1 Surgery, California Northstate University College of Medicine, Elk Grove, USA; 2 Orthopedic Surgery, Valley Consortium, Modesto, USA; 3 Ophthalmology, Lake Erie College of Osteopathic Medicine, Erie, USA; 4 Physical Medicine and Rehabilitation, California Northstate University College of Medicine, Elk Grove, USA; 5 Orthopedic Surgery, University of California Davis School of Medicine, Sacramento, USA; 6 Dermatology, California Northstate University College of Medicine, Elk Grove, USA; 7 Internal Medicine, California Northstate University College of Medicine, Elk Grove, USA; 8 Orthopedics, San Joaquin General Hospital, French Camp, USA

**Keywords:** corticosteroid injections, pain management, periprosthetic joint infection, revision surgery, total shoulder arthroplasty

## Abstract

In total joint arthroplasty, periprosthetic joint infection (PJI) can be devastating. Corticosteroid injections (CSIs) are commonly administered for temporary pain relief in the setting of various conditions. Therefore, the current systematic review aims to evaluate whether CSIs administered prior to total shoulder arthroplasty (TSA) are a risk factor for PJI and revision surgery. A search following guidelines established by the Preferred Reporting Items for Systematic Reviews and Meta-analyses was performed in the PubMed, Embase, and Scopus databases to identify studies evaluating outcomes in patients receiving CSIs prior to TSA. A meta-analysis comparing the risk of PJI between patients receiving CSIs at various time points versus those with no CSIs was performed. Seven studies comprising 49,786 patients who received CSIs were included. Most studies reported significantly higher odds of PJI and revision surgery when CSIs were administered within three months of TSA. The results of the meta-analysis found the risk of PJI was significantly higher when CSIs were administered within three months of TSA (risk ratio (RR): 1.12, 95% confidence interval (CI): 1.04-1.20, P = 0.002) but not greater than three months prior to TSA (RR: 1.02, 95% CI: 0.80-1.30, P = 0.85), relative to a control group undergoing TSA with no prior CSIs. Several studies have demonstrated that CSIs are associated with increased risks of PJI and revision surgery. A safe interval between CSI and subsequent TSA, to minimize the risk of PJI and revision surgery, appears to be around one to three months, with three months being the safest time period, as supported by the findings of our meta-analysis.

## Introduction and background

Osteoarthritis (OA) of the shoulder is a common debilitating condition affecting between 16% and 20% of patients over the age of 60 [1-4]. Total shoulder arthroplasty (TSA) is the gold-standard surgical treatment option for advanced OA, with two primary types: anatomical TSA and reverse TSA [5]. Anatomical TSA involves the replacement of the humeral head and glenoid with prosthetics, which requires intact and functional deltoid and rotator cuff muscles. Reverse TSA is conducted using a prosthesis with a fixed fulcrum, allowing the prosthetic shoulder to pivot using only the deltoid muscle, forgoing the need for functional rotator cuff muscles [6].

Corticosteroid injections (CSIs) are often provided for symptomatic relief and to reduce inflammation in the period prior to TSA [7-8]. However, evidence shows that CSIs may downregulate pro-inflammatory proteins and dampen the immune response against bacteria prone to colonizing prostheses used in arthroplasty, such as staphylococci, thus predisposing patients to PJIs when bacteria begin to multiply [9-10]. Excessive or long-term steroid use can result in decreased bone mineralization due to increased osteoclastic activity [11]. The benefits of intra-articular CSIs in providing symptom relief from OA in joints have been found to be temporally limited, as reported by recent studies [12-13]. Recent studies have reported that preoperative intra-articular CSIs administered within three to four months increase the risk of PJI in total hip and knee arthroplasty [14-19]. Another study demonstrated that administering CSIs <1 year and <1 month prior to rotator cuff repair significantly increases the risk of revision and postoperative infections, respectively [20]. Therefore, timing and the number of injections should be appropriately considered to avoid complications such as periprosthetic joint infection (PJI) or revision surgery [7].

Since both CSI and TSA are in the treatment algorithm for symptomatic shoulder OA, the primary objective of this systematic review was to evaluate whether CSIs prior to TSA result in an increased risk of PJI and revision surgery. A secondary objective was to evaluate how long before TSA CSIs are safe to administer. We hypothesized that when CSIs are administered at a shorter interval prior to TSA, there is an increased risk of PJI and the need for revision surgery.

## Review

Methods

Search Strategy

A search following guidelines established by the Preferred Reporting Items for Systematic Reviews and Meta-analyses (PRISMA) was performed in three databases on November 10, 2023: PubMed, Embase, and Scopus. Two authors identified all articles included in the study. The query was performed utilizing the Boolean search phrase “(steroid OR corticosteroid OR cortisone OR injection) AND (shoulder arthroplasty OR shoulder replacement OR total shoulder arthroplasty OR total shoulder replacement OR reverse total shoulder arthroplasty OR reverse total shoulder replacement).” There were no restrictions set to the search. Updated searches were performed periodically to identify additional new studies that may have potentially fit the inclusion criteria. Studies were included if they reported on outcomes of preoperative CSIs prior to TSA. Exclusion criteria included case reports, review articles, conference abstracts, studies performed in animals, articles not in English, expert opinions, letters to editors, and studies in which outcomes pertaining to preoperative CSIs prior to TSA were not specified. This study was registered on PROSPERO.

Study Selection

The titles and abstracts of all studies were independently reviewed by two separate reviewers using the predetermined eligibility criteria. If they were not unanimous in their decision to include or exclude a study, a third reviewer was consulted. Next, the full text of select articles was independently reviewed by two separate reviewers, and again, if the reviewers were not unanimous in their decision, a third reviewer was consulted. The reference sections of each included article were reviewed to determine whether additional studies could be added to the systematic review.

Data Extraction

Study variables extracted from each article included author, publication year, journal, level of evidence (LOE), study time period, study design, number of patients, sex, mean age, follow-up period, mean body mass index (BMI), Charlson Comorbidity Index (CCI), American Society of Anesthesiologists (ASA) score, comorbidities, type of TSA, timing of preoperative CSI, incidence of postoperative PJI, opioid use, and revision rates. All extracted data were compiled for analysis using Microsoft Word (Microsoft Office 2011; Microsoft, Redmond, WA).

Methodological Quality Assessment

The methodological quality of studies was assessed using the methodological index for non-randomized studies (MINORS) checklist. The MINORS items are scored 0 (not reported), 1 (reported but inadequate), or 2 (reported and adequate), with a maximum possible score of 16 for non-comparative studies (from eight categories) and 24 for comparative studies (from 12 categories). Two authors scored each article in the systematic review. Each author scored the article individually before reviewing their scores, and any discrepancies were resolved by re-reviewing the articles until a unanimous consensus was met.

Statistical Analysis

Descriptive statistics (mean, percentage, standard deviation, range, median) are reported in this review when applicable and when available. A meta-analysis comparing the risk of PJI between patients receiving CSIs at various time points was performed using a random effects model and a Mantel-Haenszel test. Heterogeneity was determined using the I-squared statistic. Forest plots were generated using Cochrane’s Reviewer Manager Web application (RevMan, version 5.4; The Cochrane Collaboration, London, UK).

Results

Article Selection Process

**Figure 1:**
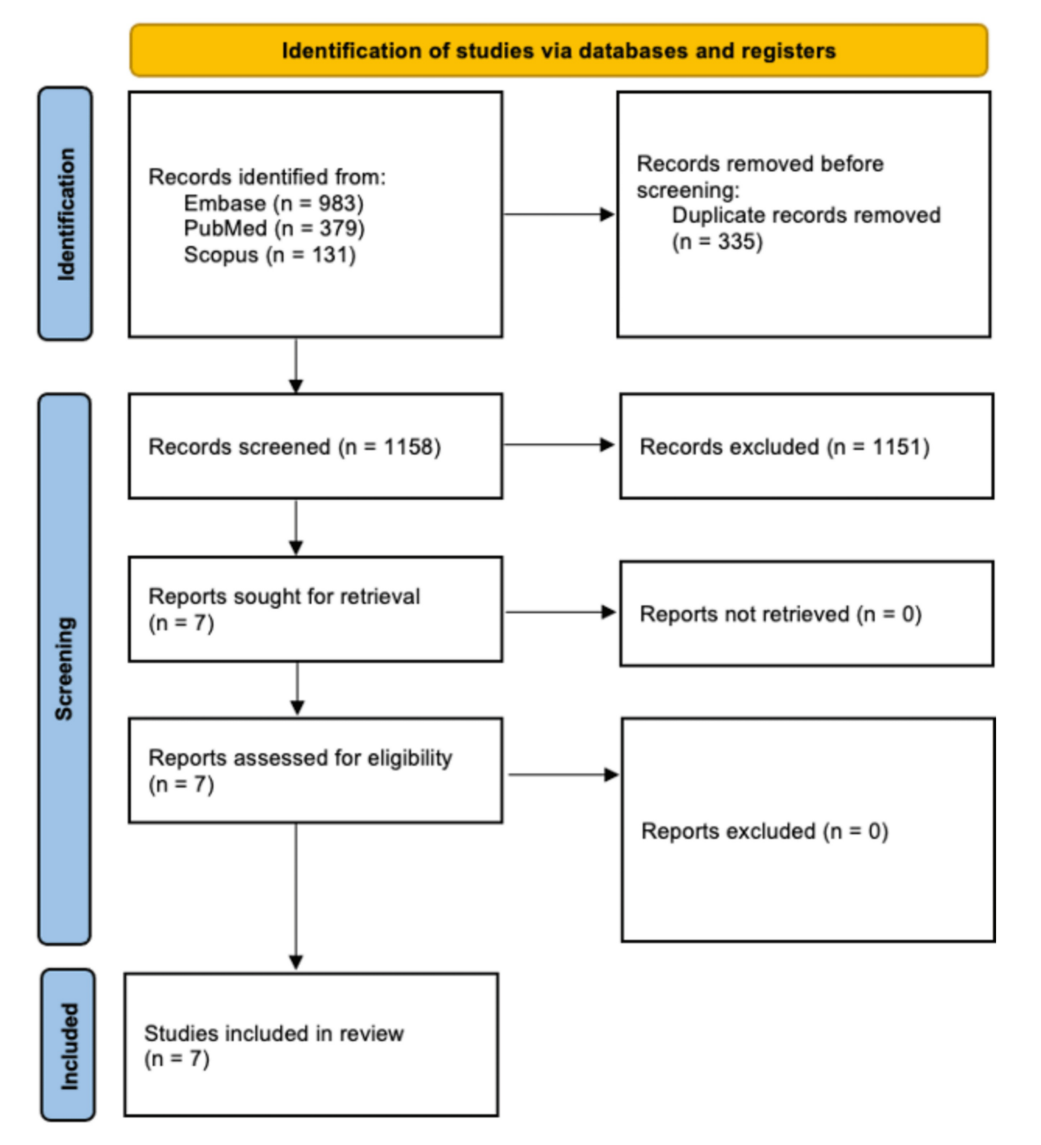
PRISMA flow diagram depicting article selection process PRISMA: Preferred Reporting Items for Systematic Reviews and Meta-analyses

Upon the initial search of the PubMed, Embase, and Cochrane Library databases, 1,493 studies were identified, of which 335 duplicates were removed. The remaining 1,158 titles and abstracts were then screened, of which 1,151 were excluded due to irrelevance to the topic of study. The remaining seven studies underwent full-text review and were all chosen to be included in this systematic review as they fit our inclusion criteria [7,21-26]. The PRISMA flow diagram depicting our search strategy and method of selecting articles is depicted in Figure 1.

Study Details and Patient Demographics

All seven included studies had a retrospective design and were all a LOE of III. The mean ± standard deviation (range) of the MINORS score was 16.7 ± 1.0 (15-18). Five of the studies utilized data derived from medical insurance claims databases, whereas two studies used data from a single institution. Four studies reported on outcomes of TSA [21,23-25], one reported on outcomes of rTSA [22], and two reported on outcomes of both TSA and rTSA [7,26]. Four studies compared the outcomes of patients with preoperative CSI at various time points to those without preoperative CSI [7,21-23]. One study compared outcomes of one and two or more preoperative CSIs administered within a year prior to TSA with no preoperative CSI [24]. The two studies from single institutions compared outcomes of a group who received preoperative CSIs at a mean of 11.4 months (range: 2.5-172.5) and 4.7 ± 2.7 (0.9-12) months, respectively, before TSA with those who received no preoperative CSI [25-26].

Across all seven studies, there were a total of 49,786 patients in whom a preoperative CSI was administered and 85,974 patients in whom a preoperative CSI was not administered. The definition of no preoperative CSI, however, varied between studies. Two studies defined it as no CSI for at least six months prior to TSA [21-22]. Three studies defined it as no CSI for at least a year prior to TSA [7,24,26]. Two studies defined it as no prior documented CSI [23,25]. Comorbidities of patients were described using the CCI in three studies [7,21-22] or ASA score in one study [25]. When comparing complications, PJI, and revision between injection and no-injection groups, both groups were adjusted for demographic variables and comorbidities in five studies and only comorbidities in one study [26]. Rashid et al. [25] did not report whether they adjusted for demographic variables and comorbidities. Table 1 summarizes the study characteristics and demographics of patients.

**Table 1:** Study characteristics and patient demographics CSI: corticosteroid injection, NR: not reported, M/F: male/female, MINORS: methodological index for non-randomized studies

Author	MINORS	Number of patients (M/F)		Age (years)		Comorbidities	
		CSI	No CSI	CSI	No CSI	CSI	No CSI
Baksh et al., 2023 [21]	16	<1 month: 214 (86/128); 1-2 month: 473 (191/282); 2-3 month: 604 (273/331)	15486 (7949/7537)	<1 month: 66 ± 9.3; 1-2 month: 66 ± 9.3; 2-3 month: 66 ± 9.1	66 ± 9.6	CCI >3 <1 month: 32; 1-2 month: 76; 2-3 month: 94	CCI >3 3582
Baksh et al., 2023 [22]	16	<1 month: 5607 (2053/3554); 1-2 month: 3024 (1154/1870); 2-3 month: 1572 (606/966); >3 month: 16302 (6171/10131)	21938 (9269/12669)	<1 month: 69 ± 8.12; 1-2 month: 69 ± 8.3; 2-3 month: 69 ± 7.88; >3 month: 68 ± 8.12	67 ± 8.26	CCI >3 <1 month: 1224; 1-2 month: 580; 2-3 month: 302; >3 month: 2459	CCI >3 2874
Werner et al., 2016 [23]	18	<3 month: 636 (221/415); 3-12 month: 1573 (526/1047)	6211 (2146/4065)	<3 month <70: 174; 70-80: 312; >80 154	<70: 1631; 70-80: 3042; >80: 1538	CCI similar for both groups (P > 0.05)	
				3-12 month <70: 403; 70-80: 772; >80: 398			
Lemme et al., 2023 [24]	17	1 CSI at <3 month: 12397; 2+ CSI at <3 month: 5577	39563	NR	NR	NR	NR
Rashid et al., 2015 [25]	15	23 (5/18)	60 (16/44)	73 (54-90)	75 (34-90)	ASA: 2*	ASA: 2*
Stadecker et al., 2022 [7]	18	<3 month: 250; 3-6 month: 801; 6-9 month: 410; 9-12 month: 171	2620 (1267/1353)	<65: 250; 65-69: 576; 70-74: 682; 75-79: 555; 80-84: 428; >84: 129	<65: 95; 65-69: 336; 70-74: 400; 75-79: 477; 80-84: 240; >84: 84	CCI 0: 311; 1: 354; 2: 211; 3: 201; >3: 555	CCI 0: 915; 1: 354; 2: 211; 3: 201; >3: 476
Cooper et al., 2024 [26]	17	134 (57/77)	96 (51/45)	68.9 ± 7.7	66 ± 8.7	CCI similar for both groups (P > 0.05)	

Summary of Study Findings

Three of the seven studies found that a shorter interval between a preoperative CSI was associated with higher odds of PJI [21-23]. Baksh et al. [21], in their study analyzing TSA, found that a CSI administered <1 month prior to TSA was associated with greater odds of PJI at both one (P = 0.007) and two years (P = 0.016). However, a CSI administered one to two or two to three months prior to TSA was not associated with greater odds of PJI at both one- and two-year follow-ups (P > 0.05). Baksh et al. [22], in their study analyzing rTSA, found that a CSI administered <1 month prior to rTSA was associated with greater odds of PJI at three months (P < 0.001) and one year (P = 0.015) but not at two years. However, a CSI administered 1-2, 2-3, and >3 months prior to rTSA was not associated with greater odds of PJI at three months, one year, and two years (P > 0.05). Finally, Werner et al. [23] found that administering a CSI <3 months prior to TSA was associated with greater odds of PJI at both three (P = 0.007) and six months (P = 0.001). However, when administered 3-12 months prior to TSA, the odds of PJI were similar to having administered no preoperative CSI (P > 0.05).

Stadecker et al. [7] found that a shorter interval between a preoperative CSI was associated with higher odds of revision surgery. Patients in which a CSI was administered <3 months prior to TSA had significantly higher odds of revision (P < 0.001) at two years, whereas those in which a CSI was administered 3-6, 6-9, and 9-12 months prior to TSA did not have significantly greater odds of revision (P > 0.05). Additionally, a subanalysis of patients who required revision surgery found that greater than 25% of the patients (n = 17) undergoing revision were less than 65 years of age, despite this age cohort representing less than 10% of the entire study cohort.

Lemme et al. [24] found that higher odds of revision at six months were associated with both one (P < 0.0001) and two or more (P = 0.02) preoperative CSIs administered <3 months prior to TSA. A higher odds of revision at one year was also associated with both one (P < 0.0001) and two or more (P = 0.002) preoperative CSIs. However, higher odds of PJI at one year were not associated with one (P = 0.2082) and two or more (P = 0.8838) preoperative CSIs. Additionally, relative to the group with no preoperative CSI, patients with one preoperative CSI were at increased risk (P < 0.05) of filling an opioid prescription at one, six, and 12 months. Those with two or more preoperative CSIs were at even higher risk (P < 0.01) of filling an opioid prescription at one, three, six, and 12 months.

Rashid et al. [25] found no significant relationship between preoperative CSIs administered at a mean of 11.4 months (range: 2.5-172.5) before TSA and the development of a superficial or deep surgical site infection. Patients in which a preoperative CSI was administered were followed for a mean of 16.6 months (range: 3.2-53.3), and those in which a preoperative CSI was not administered were followed for a mean of 20.1 months (range: 1.6-67.4).

Cooper et al. [26] found no significant differences in PJI, instability, and revision surgery between patients receiving CSIs at a mean of 4.7 ± 2.7 (0.9-12) months prior to TSA versus not receiving CSIs. At a three-year follow-up, they did find that patients receiving CSIs had significantly higher ASES (82 vs. 76, p<0.01) and SANE (70 vs. 63, p<0.01) scores. However, other PROs, including the VAS, VR12 PCS, and VR12 MCS, were not significantly different between both groups.

Table 2 summarizes the incidence of complications, PJI, and revision for patients in each study.

**Table 2:** Incidence of complications and revision surgery OR (95% CI) is relative to the group with no CSI in each study. Significant p-values (P < 0.05) are bolded. CSI: corticosteroid injection, PJI: periprosthetic joint infection, NR: not reported, SSSI: superficial surgical site infection, DSSI: deep surgical site infection, CCI: Charlson Comorbidity Index, OR (95% CI): odds ratio (95% confidence interval)

Author	Complications (n (%))		Revision (n (%))		Statistical findings of individual studies			
	CSI	No CSI	CSI	No CSI				
Baksh et al., 2023 [21]	PJI at 3 months <1 month: <11 (<5%); 1-2 month: <11 (<2.3%); 2-3 month: <11 (<1.8%)	PJI at 3 months: 260 (1.7%)	NR	NR	OR (95% CI) of PJI at 1 year <1 month: 2.29 (1.2-4; P = 0.007); 1-2 month: 0.97 (0.5-1.7; P = 0.927); 2-3 month: 0.94 (0.5-1.6; P = 0.830)		OR (95% CI) of PJI at 2 years <1 month: 2.03 (1.1-3.5; P = 0.016); 1-2 month: 1.24 (0.7-2: P = 0.296); 2-3 month: 1.06 (0.6-1.7; P = 0.807)	
	PJI at 1 year <1 month: 12 (5.6%); 1-2 month: 11 (2.3%); 2-3 month: 14 (2.3%)	PJI at 1 year: 383 (2.5%)						
	PJI at 2 years <1 month: 13 (6.1%); 1-2 month: 17 (3.6%); 2-3 month: 19 (3.1%)	PJI at 2 year: 461 (3%)						
Baksh et al., 2023 [22]	PJI at 3 months <1 month: 529 (9.4%); 1-2 month: 232 (7.7%); 2-3 month: 134 (8.5%); >3 month: 1373 (8.4%)	PJI at 3 months: 1588 (7.2%)	NR	NR	OR (95% CI) of PJI at 90 days <1 month: 1.23 (1.1-1.4; P < 0.001); 1-2 month: 1.03 (0.9-1.2; P = 0.723); 2-3 month: 1.18 (1-1.4; P = 0.088); >3 month: 1.09 (0.9-1.3; P = 0.278)			
	PJI at 1 year <1 month: 556 (9.9%); 1-2 month: 256 (8.5%); 2-3 month: 145 (9.2%); >3 month: 1495 (9.2%)	PJI at 1 year: 1791 (8.2%)			OR (95% CI) of PJI at 1 year <1 month: 1.14 (1-1.3; P = 0.015); 1-2 month: 1 (0.9-1.2; P = 0.998); 2-3 month: 1.12 (0.9-1.3; P = 0.212); >3 month: 1.09 (0.9-1.3; P = 0.26)			
	PJI at 2 year <1 month: 564 (10.1%); 1-2 month: 265 (8.8%); 2-3 month: 151 (9.6%); >3 month: 1537 (9.4%)	PJI at 2 years: 1877 (8.6%)			OR (95% CI) of PJI at 2 years <1 month: 1.09 (1-1.2; P = 0.088); 1-2 month: 0.98 (0.9-1.1; P = 0.825); 2-3 month: 1.11 (0.9-1.3; P = 0.237); >3 month: 1.07 (0.9-1.2; P = 0.375)			
Werner et al., 2016 [23]	PJI at 3 months <3 month: 19 (3%); 3-12 month: 29 (4.6%)	PJI at 3 months: 92 (1.5%)	NR	NR	OR (95% CI) of PJI at 3 months <3 month: 2 (1.2-3.4; P = 0.007); 3-12 month: 0.7 (0.4-1.2; P = 0.199)			
	PJI at 6 months <3 month: 16 (1%); 3-12 month: 31 (2%)	PJI at 6 months: 147 (2.4%)			OR (95% CI) of PJI at 6 months <3 month: 2 (1.3-3; P = 0.001); 3-12 month: 0.8 (0.6-1.2; P = 0.399)			
Lemme et al., 2023 [24]	PJI at 1 year 1 CSI at <3 month: 36 (0.3%); 2+ CSI at <3 month: 20 (0.4%)	PJI at 1 year: 137 (0.3%)	Revision at 6 months 1 CSI: 152 (1.2%); 2+ CSI: 61 (1.1%)	Revision at 6 months: 311 (0.8%)	OR (95% CI) of PJI at 1 year 1 CSI: 0.79 (0.5-1.1; P = 0.2082); 2+ CSI: 0.97 (0.6-1.5; P = 0.8838)	OR (95% CI) of revision at 6 months 1 CSI: 1.56 (1.2-1.9; P < 0.0001); 2+ CSI: 1.39 (1-1.8; P = 0.02)		OR (95% CI) of revision at 1 year 1 CSI: 1.53 (1.3-1.8; P < 0.0001); 2+ CSI: 1.44 (1.1-1.8; P = 0.002)
			Revision at 1 year 1 CSI: 210 (1.7%); 2+ CSI: 89 (1.6%)	Revision at 1 year: 441 (1.1%)				
Rashid et al., 2015 [25]	SSSI: 0 DSSI: 1 (4.3%)	SSSI: 0; DSSI: 0	NR	NR	No significant differences in infection rates (P > 0.05)			
Stadecker et al., 2022 [7]	% Revisions due to PJI at 2 years <3 month: 71.4%; 3-6 month: 60%; 6-9 month: 50%; 9-12 month: 33.3%	NR	Revision at 2 years <3 month: 14 (5.6%); 3-6 month: <11 (<1.4%); 6-9 month: <11 (<2.7%); 9-12 month: <11 (<6.4%)	Revision at 2 years: 30 (1.1%)	The OR (95% CI) of revision at 2 years was 2.61 (1.8-3.3; P < 0.001) for the <3 month group. The 3-6, 6-9, and 9-12 month groups did not have significantly higher odds of revision			
					Multivariate analysis of risk factors associated with significantly higher odds of revision: CCI > 3 (4 (1.4-8.9; P = 0.036)), age > 84 (1.33 (1.1-1.6; P = 0.028)), BMI ≥30 kg/m^2^ (1.56 (1.2-1.8; P = 0.011)), and smoking (1.3 (1.1-1.5; P = 0.024). The comparison group included no injection, female sex, CCI of 0, age < 65, nonobese (BMI <30 kg/m^2^), and no smoking			
Cooper et al., 2024 [27]	PJI: 2 (1.5%); instability: 4 (3%); other: 2 (1.5%)	PJI: 2 (2.1%); instability: 0; other: 0	4 (3%)	1 (1%)	No significant differences in complication or revision rates (P = 0.435)			

Meta-analysis Evaluating Risk of PJI

**Figure 2:**
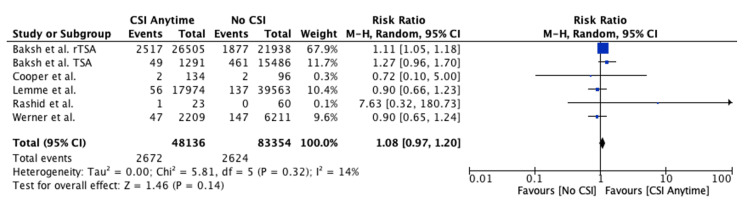
Forest plot evaluating risk of PJI when CSIs are administered anytime prior to TSA Baksh et al., 2023, rTSA [22]; Baksh et al., 2023, TSA [21]; Cooper et al., 2024 [26], Lemme et al., 2023 [24]; Rashid et al., 2015 [25]; Werner et al., 2016 [23] PJI: periprosthetic joint infection, CSIs: corticosteroid injections, TSA: total shoulder arthroplasty

CSI anytime prior to TSA: The risk of PJI when CSIs were administered anytime prior to TSA was similar to the group receiving no prior CSI (risk ratio (RR): 1.08, 95% confidence interval (CI): 0.97-1.20, P = 0.14) (Figure 2).

**Figure 3:**
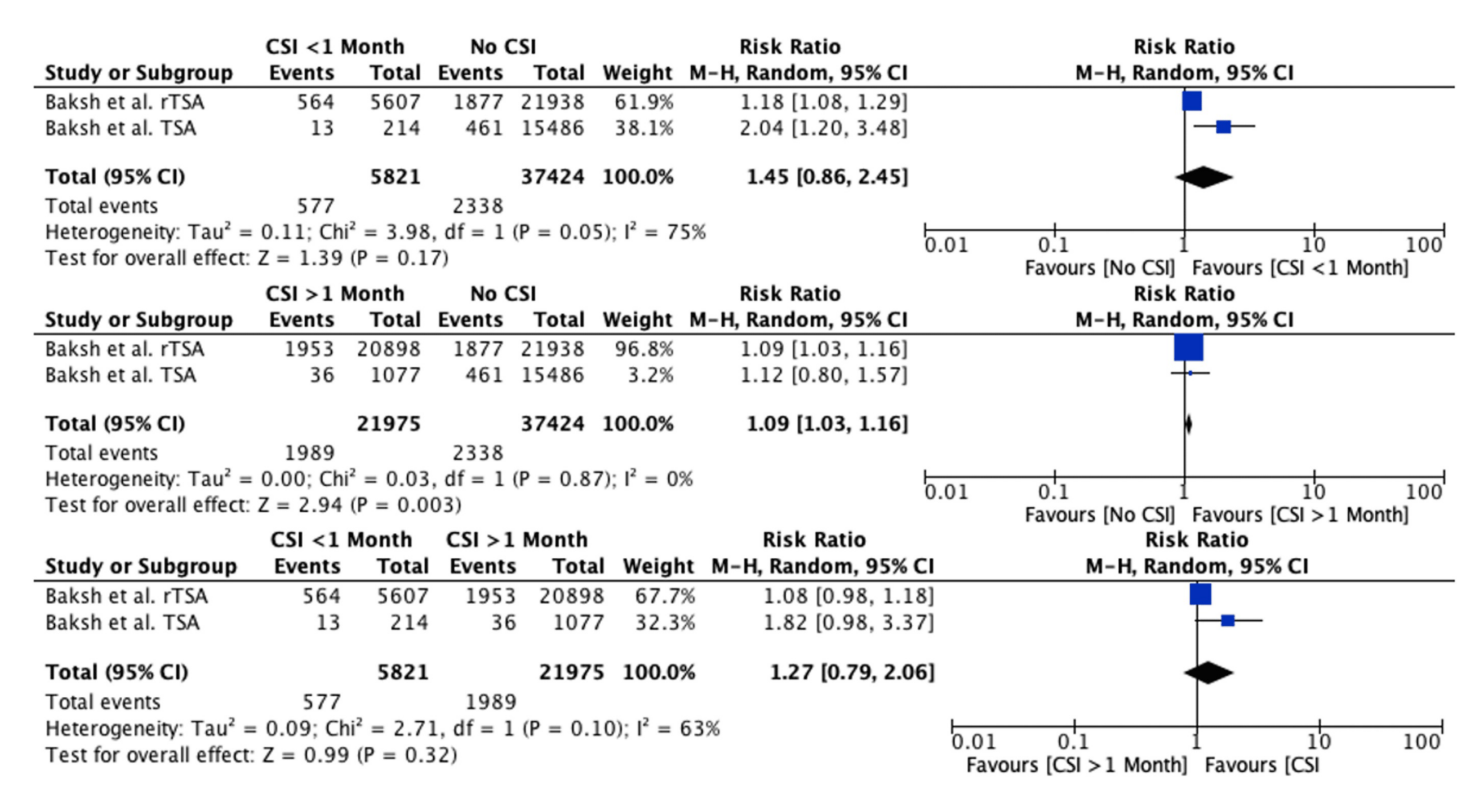
Forest plot evaluating the risk of PJI with one month as a cutoff time point Baksh et al., 2023, rTSA [22]; Baksh et al., 2023, TSA [21] PJI: periprosthetic joint infection, TSA: total shoulder arthroplasty

CSI within or greater than one month prior to TSA: The risk of PJI was significantly higher in patients receiving CSIs greater than one month prior to TSA compared to patients receiving no prior CSI (RR: 1.09, 95% CI: 1.03-1.16, P = 0.003). The risk of PJI was similar when CSIs were administered within one month prior to TSA compared to patients receiving no CSI (RR: 1.45, 95% CI: 0.86-2.45, P = 0.17) and receiving CSIs greater than one month prior to TSA (RR: 1.27, 95% CI: 0.79-2.06, P = 0.32) (Figure 3).

**Figure 4:**
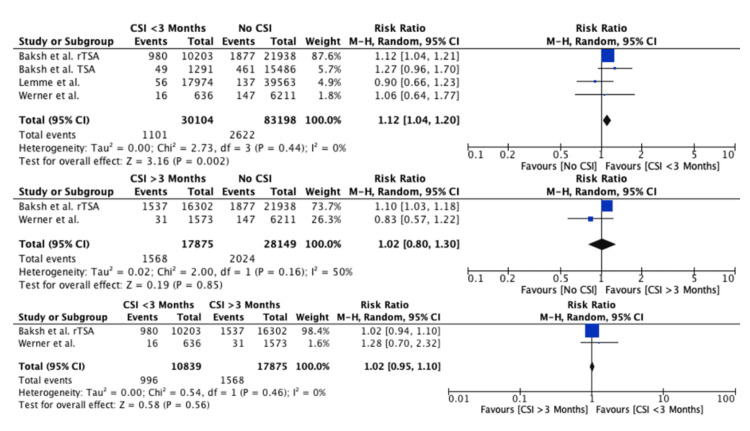
Forest plot evaluating the risk of PJI with three months as a cutoff time point Baksh et al., 2023, rTSA [22]; Baksh et al., 2023, TSA [21]; Lemme et al., 2023 [24]; Werner et al., 2016 [23] PJI: periprosthetic joint infection, TSA: total shoulder arthroplasty

CSI within or greater than three months prior to TSA: The risk of PJI was significantly higher in patients receiving CSIs within three months prior to TSA compared to patients receiving no prior CSI (RR: 1.12, 95% CI: 1.04-1.20, P = 0.002). The risk of PJI was similar when CSIs were administered greater than three months prior to TSA compared to patients receiving no CSI (RR: 1.02, 95% CI: 0.80-1.30, P = 0.85) and receiving CSIs within three months prior to TSA (RR: 1.02, 95% CI: 0.95-1.10, P = 0.56) (Figure 4).

Discussion

This systematic review analyzed seven studies comparing the administration of preoperative CSI at various time points or the number of preoperative CSIs to control groups in which preoperative CSIs were not administered. The individual studies reported a higher risk of PJI and revision surgery when CSIs were administered within one to three months prior to TSA but not beyond three months. In our meta-analysis, we evaluated the risk of PJI with one and three months as cutoff time points; however, our results were conflicting. CSIs within one month were not significantly associated with an increased risk of PJI, but CSIs administered greater than one month were. Conversely, CSIs within three months were significantly associated with an increased risk of PJI, but CSIs administered beyond three months were not. While the meta-analysis results with one month as a time point cutoff were conflicting, the results at three months concur with those of other meta-analyses evaluating the risk of PJI in TKA and THA [14,18].

In our meta-analysis, the study by Baksh et al. [22] made up the majority of the weight, which may have skewed the results. As part of a sub-analysis for forest plots, which included data from the Baksh et al. [22] study in addition to at least two other studies, we re-performed the analysis after removing data from the Baksh et al. [22] study to evaluate whether the results were consistent or not. This was the case for the two meta-analyses comparing the risk of PJI when CSIs were administered anytime prior (Figure 2) and within three months (Figure 4) compared to no CSI. For CSIs administered anytime prior versus no CSI, the PJI risk remained insignificant (RR: 1.03, 95% CI: 0.82-1.28, P = 0.83). However, for CSIs administered within three months compared to no CSI, the risk of PJI was now found not to be significantly different between both groups (RR: 1.08, 95% CI: 0.86-1.36, P = 0.52), while previously, as demonstrated in Figure 4, the risk of PJI was significantly higher in the CSIs within the three-month group. Therefore, making a clear-cut recommendation regarding the safest time interval between CSI and subsequent TSA remains difficult.

Though one study found only a higher odds of revision when a CSI was administered <3 months prior to TSA and not when administered 3-6, 6-9, and 9-12 months prior, another study found that even if a single CSI was administered within a whole year prior to TSA, there were significantly greater odds of requiring revision TSA. With only two studies reporting on the association between preoperative CSIs and the need for future revision surgery, and both with conflicting results, along with no meta-analysis on the risk of revision, it is also difficult to make clear-cut recommendations on the timing of preoperative CSIs to avoid the risk of requiring revision surgery.

Two studies [25-26] included in our systematic review did not match patients based on both demographic variables and baseline comorbidities. The meta-analysis in Figure 2, evaluating the risk of PJI when CSIs were administered anytime prior to TSA compared to no CSI, included both of these studies, so we performed a sub-analysis by removing the data from these studies. Compared to the original finding of a similar risk of PJI between both groups (RR: 1.08, 95% CI: 0.97-1.20, P = 0.14), we found that the risk of PJI to remain similar between both groups (RR: 1.08, 95% CI: 0.95-1.21, P = 0.23).

Compared to patients who did not receive any CSIs, there was a significantly higher risk of PJI when CSIs were administered within three months of TSA (P = 0.002) but not when administered greater than three months of TSA (P = 0.85). Additionally, when the within versus greater than three-month groups were directly compared, there was no significant difference in the risk of PJI (P = 0.56). Two previous meta-analyses in total hip and knee arthroplasty similarly found that among patients receiving CSIs 0-3, 4-6, and 7-12 months prior to surgery, significantly higher postoperative PJI rates were seen in the 0-3 months group (P = 0.03 and P < 0.00001), but not the 4-6 (P = 0.13 and P = 0.24) and 7-12 (P = 0.6) months group [14,18]. Therefore, higher PJI rates when CSIs are administered within three months appear to be consistent across TSA, THA, and TKA.

Only one study in our review evaluated the effect of the number of preoperative CSIs on postoperative PJI and revision rates. The study found a similar rate of PJI between control and 1 or 2+ CSIs within three months prior to TSA. However, revision rates were significantly higher in both the 1 and 2+ CSI groups. There is a paucity of literature regarding the effect of multiple CSIs on the risk of PJI and revision surgery in other joints, with only one study on TKAs that concluded that multiple CSIs could safely be administered more than three months prior to TKA without an increased risk of PJI [27]. However, there has been no report regarding the effect of multiple CSIs on revision rates in other joints.

The findings of this systematic review should be interpreted in the context of its limitations. First, all studies were retrospective in their designs and thus had a lower LOE than would a prospective study; however, given the nature of the research question, a prospective or randomized design would likely not have been feasible. Second, some studies included in this review used data from national insurance databases that rely on accurate coding of providers, an aspect that cannot be verified and, therefore, not controlled. Third, with data derived from national insurance databases, there could possibly have been an overlap between studies that inflated the total number of patients and instances of PJI and revision surgery.

## Conclusions

Several studies have demonstrated that CSIs are associated with increased risks of PJI and revision surgery. The individual studies reported that a safe interval between CSI and subsequent TSA to minimize the risk of PJI and revision surgery appears to be around one to three months, with three months being the safest time period, as supported by the findings of our meta-analysis.
